# Targeting gene expression to specific cells of kidney tubules in vivo, using adenoviral promoter fragments

**DOI:** 10.1371/journal.pone.0168638

**Published:** 2017-03-02

**Authors:** Sumiyo Watanabe, Toru Ogasawara, Yoshifuru Tamura, Taku Saito, Toshiyuki Ikeda, Nobuchika Suzuki, Tatsuo Shimosawa, Shigeru Shibata, Ung-il Chung, Masaomi Nangaku, Shunya Uchida

**Affiliations:** 1 Center for Disease Biology and Integrative Medicine, Faculty of Medicine, The University of Tokyo, Hongo Bunkyo-ku, Tokyo, Japan; 2 Division of Nephrology and Endocrinology, The University of Tokyo, Graduate School of Medicine, Hongo, Bunkyo-ku, Tokyo, Japan; 3 Department of Internal Medicine, Teikyo University School of Medicine, Kaga, Itabashi-ku, Tokyo, Japan; 4 Department of Oral and Maxillofacial Surgery, Faculty of Medicine, The University of Tokyo, Hongo, Bunkyo-Ku, Tokyo, Japan; 5 Department of Orthopaedic Surgery, Faculty of Medicine, The University of Tokyo, Japan; 6 Department of Blood Transfusion, Faculty of Medicine, The University of Tokyo, Japan; 7 Department of Bioregulation, Nippon Medical School, Sendagi, Bunkyo-ku, Tokyo, Japan; University of Louisville, UNITED STATES

## Abstract

Although techniques for cell-specific gene expression via viral transfer have advanced, many challenges (e.g., viral vector design, transduction of genes into specific target cells) still remain. We investigated a novel, simple methodology for using adenovirus transfer to target specific cells of the kidney tubules for the expression of exogenous proteins. We selected genes encoding sodium-dependent phosphate transporter type 2a (NPT2a) in the proximal tubule, sodium-potassium-2-chloride cotransporter (NKCC2) in the thick ascending limb of Henle (TALH), and aquaporin 2 (AQP2) in the collecting duct. The promoters of the three genes were linked to a GFP-coding fragment, the final constructs were then incorporated into an adenovirus vector, and this was then used to generate gene-manipulated viruses. After flushing circulating blood, viruses were directly injected into the renal arteries of rats and were allowed to site-specifically expression in tubule cells, and rats were then euthanized to obtain kidney tissues for immunohistochemistry. Double staining with adenovirus-derived EGFP and endogenous proteins were examined to verify orthotopic expression, i.e. “adenovirus driven NPT2a-EGFP and endogenous NHE3 protein”, “adenovirus driven NKCC2-EGFP and endogenous NKCC2 protein” and “adenovirus driven AQP2-EGFP and endogenous AQP2 protein”. Owing to a lack of finding good working anti-NPT2a antibody, an antibody against a different protein (sodium-hydrogen exchanger 3 or NHE3) that is also specifically expressed in the proximal tubule was used. Kidney structures were well-preserved, and other organ tissues did not show EGFP staining. Our gene transfer method is easier than using genetically engineered animals, and it confers the advantage of allowing the manipulation of gene transfer after birth. This is the first method to successfully target gene expression to specific cells in the kidney tubules. This study may serve as the first step for safe and effective gene therapy in the kidney tubule diseases.

## Introduction

The prevalence of chronic kidney disease (CKD) is estimated to be 8–16% of the global population [1], and the presence of CKD is intimately associated with large increases in both end-stage kidney disease and cardiovascular disease risk [2]. One major cause of CKD is the disruption of genes that are expressed in specific cells of the renal tubules; this can result in several different diseases, e.g., Fanconi syndrome, Bartter syndrome, Gitelman syndrome, and Dent disease [3, 4, 5, 6]. Although gene transduction may be useful to treat these patients, kidneys are composed of many distinct cell types; therefore, highly accurate, site-specific gene expression is necessary to minimize adverse effects resulting from such a treatment [7]. Furthermore, gene therapy has not yet been established in nephrology [8].

From the standpoint of gene transduction efficiency, adenoviral vectors are the strongest among all available vectors, including other viral and artificial vectors [9]; adenovirus vectors are the most commonly used viral vectors in gene therapy clinical trials (23.3%) [10]. However, systemic administration of adenoviruses results in gene expression exclusively in the liver [11–18]. To overcome the mass accumulation of adenoviruses in the liver, two approaches have been conducted to date [19]. One approach is to modify the existing viruses to develop artificial transduction vehicles; however, many of these are also accumulated in the liver [20, 21]. Another approach is direct administration to the target organ. However, direct administration into deep tissues is invasive, and it is difficult to induce genes in the entire tissues, or at the desired sites, of the target organ. For these reasons, targeted gene expression is currently limited to the surface tissues of such organs as the eyes and the respiratory tract. In the case of the kidney and urinary tract, the target sites have been the bladder [21, 22], kidney parenchyma [14, 22], and *ex vivo* organs during surgery [7, 16], which were used in an attempt to obtain direct access to tissues. However, non-specific CAG promoters are expressed not only in the target cells, but also in other types of cells located within the injection area; furthermore, direct access to target organs via surgery may carry high risks of complications due to infection, bleeding, and tissue damage. In addition, direct surgical access to a target organ does not facilitate the selection of specific cell types, because most organs contain many different types of cells within close proximity.

Therefore, we were prompted to achieve transgene expression in specific cell types within the kidneys. Briefly, we designed and cloned several promoters and incorporated to adenovirus vectors to make express EGFP into the kidneys that should be cell-specific, so as to circumvent expression of EGFP in non-targeted cells. The cloning promoters were for the very site-specific expression genes within three different segments of the kidneys—the proximal tubule, the thick ascending limb of Henle (TALH), and the collecting duct. Then, the renal artery flushed out with natural saline, these adenovirus vectors are injected into the renal artery, the renal artery was clamped for a while to enhance infection efficiency, and the last, the clamps were removed from the vessels to restore blood flow in rats.

The main goal of this study was to create a safe, effective method for accomplishing targeted gene expression in the kidneys. Here, we show that combining site-specific promoters and adenoviral transduction methodology allows the expression of transgenes specifically to the TALH, the proximal tubules, and the distal tubules, without causing kidney damage, in rats.

## Materials and methods

### Gene analyses

We focused on representative genes normally expressed in specific kidney tubule structures: sodium-dependent phosphate transporter 2 (NPT2a; SLC34A1) in the proximal tubule [15], sodium-potassium chloride cotransporter (NKCC2; SLC12A1) in the TALH [16], and aquaporin 2 (AQP2) in the collecting duct or distal tubule [16]. Genome sequences of these three genes were examined using National Center for Biotechnology Information (NCBI) databases and the Ensembl Genome Browser website (http://www.ensembl.org/index.html). The enzyme cutting sites for cloning were examined using the Mikenora free software (Daiich Kagaku, Akashi, Japan). To match the genome sequences among many species, and to determine the transcription factor binding sites, Vector NTI Advance software (version 10, Invitrogen, Carlsbad, CA) and the TRANSFAC database with "Match" application (http://www.gene-regulation.com/pub/programs.html) were used. The gene accession numbers of the 5′ upstream sequences of *Npt2a*, *Nkcc2*, and *Aqp2* are ENSMUSG 00000027202, ENSRNOG 00000015262, and ENSRNOG 00000000297, respectively.

### Promoter construct cloning

#### Npt2a promoter

The genomic clone was used as a template for PCR amplification, with a sense primer at position -3,379 (5′-AAATTTGTCTGGATATAATGT-3′) and an anti-sense primer at position +630 (5′-GATGGGCACCCACAATGAGTCCTGTGTAT-3′), with "-1" being relative to the ATG translational start codon. We tried to incorporate the closest sequence (-1) just upstream of the ATG translation starting codon to make the total length as long as possible (up to ~4 kbp). PCR conditions were the following: 98°C for 5 min, 66°C for 30 sec, 68°C for 4 min and 30 sec for 30 cycles, and 68°C for 10 min.

#### Nkcc2 promoter

We examined vectors as previously published by Dr. Peter Igarashi [17]. They two different, constructed proximal 5′-flanking region segments of the *Nkcc2* gene constructs were cloned from PGL3 basic vectors (Promega, Madison, WI); one spanned positions -2,255 to -188 (construct named pNKCC2DI), and the other ran from positions -1,529 to -188 (construct named pNKCC2pvu). We also constructed a third promoter to be of a length intermediate between pNKCC2DI and pNKCC2pvu. The genomic clone was used as a template for PCR amplification, with a sense primer located at position -1,612 (5′- GCAATTAGCAAGGCAAGACAGTT-3′) and an anti-sense primer ending at position +9 (5′- CTCCCATGCAAACTTCAGAATG-3′). PCR conditions were the following: 95°C for 3 min, 62°C for 1 min, 68°C for 1 min and 40 sec for 30 cycles, and 68°C for 10 min.

#### Aqp2 promoter

We constructed plasmids carrying three different sections of the 5′ upstream region of *Aqp2*, because of the long promoter sequence. The first construct spanned from positions -8,347 (5′-TCTTACCATCTATTAAAGACAAAAGCAA-3′) to -2,936 (5′-CTTCTGGTATTAACAAATCTGATCTCTG-3′). PCR was performed as follows: 98°C for 5 min, 66°C for 30 sec, 68°C for 5 min for 30 cycles, and 68°C for 10 min. The second construct spanned from position -3,652 (5′-TGATCCAATTGATAAGATATAATTTTTGC-3′) to position +1,860 (5′-TATACCATGTTAGAAAGTTTGATTTTGG-3′). PCR was performed as follows: 98°C for 30 sec, 66°C for 30 sec, 68°C for 4 min and 30 sec for 30 cycles, and 68°C for 10 min. The third construct spanned from positions +906 to +930. We then adopted the oligo construct spanning from -5,338 to +930 (-1 relative to the ATG translational start codon) (5′- AGAAGTCGGAGCAGCACCGGTTCA -3′) as the AQP2 promoter. The three *Aqp2* constructs were ligated into one fragment, which had a sequence that spanned from position -8,347 to -1 from the starting codon.

#### CAG (non-specific) positive control promoter

The enhanced green fluorescent protein (EGFP) gene in the pEGFPN1 vector was subcloned into the CAG-promoter cosmid (pAxCwit adenovirus cosmid) (Takara Bio, Otsu, Japan).

### Adenovirus construction

The promoters of the test genes (*Npt2a*, *Nkcc2*, and *Aqp2*) were purified from genomic DNA obtained from the blood of Wistar rats. Rats were purchased from the Charles River Laboratory in Japan. The gene constructs of pNKCC2DI and pNKCC2pvu were gifts from Dr. Peter Igarashi (University of Texas Southwestern Medical Center). All promoters created for this study are available upon request. Of note is that the adenovirus construct lacks the coding region of translation.

Adenovirus constructs were subcloned into the Zero Blunt cloning vector (Invitrogen k2700-20, Carlsbad, CA), then were subcloned into the pEGFPN1 vector (Clontech, Mountain View, CA), and were finally subcloned into the promoterless pAxCwit adenovirus cosmid. NKCC2DI-EGFP, NKCC2pvu-EGFP, pNKCC2-EGFP, NPT2a-EGFP, and AQP2 EGFP were used to infect human embryonic kidney cells (HEK293 cells; ATCC, Manassas, VA), which were cultured with Dulbecco’s modified Eagle’s medium (DMEM; Nissui, Tokyo, Japan) containing 5% fetal bovine serum. We precisely selected a single clone of adenovirus, and identified its DNA via enzyme cutting and DNA sequence analyses. The titer of each adenovirus exceeded 10^13^ pfu/mL. After purification of these adenoviruses, each colony was used to infect four rats; CAG-EGFP was used to infect three rats

### Adenoviral infection procedure

When adenoviruses are introduced into a target organ via peripheral intravenous or intra-arterial routes, co-expression in the liver cells is almost always observed [10]. For selective infection of the kidneys, uptake of the adenovirus into kidney cells must occur before the adenovirus arrives at the liver. We used four different approaches for injecting adenovirus into the kidney of 8- to 10-week-old rats (S1 Fig): (a) intra-arterial bolus injection into the renal artery for 2 min, (b) continuous intra-arterial slow drips into the renal artery for 16 h, (c) an injection into the bladder, to be transduced by retrograde infusion, and (d) 10 repetitions of direct injections into the renal parenchyma from all directions. To describe approach (a) in greater detail: following clamping between the abdominal aorta and the beginning of the left renal artery, 1 mL of normal saline was injected into the left renal artery over 2 min. Then, 1 mL of adenovirus solution was injected into the artery over 2 min, followed by clamping of the renal vein for 10 min, after which the clips were removed from the vessels to restore renal blood flow. Surgery was performed under nitrous oxide-oxygen anesthesia. All experimental procedures were approved by the University of Tokyo Animal Care Committee # KA10-2-1. 43 rats were used for this study.

### Immunohistochemistry

Four days after injection, we anesthetized and euthanized the gene-transferred rats. Each removed kidney was fixed with 4% paraformaldehyde for two nights, after which it was paraffin-embedded and prepared in sections. Deparaffinized sections were stained with HRP (1:200; Dako, Denmark and Carpinteria, CA, USA)-DAB (Dako) and ALP (1:80; Dako)-NBT-BCIP (Dako). We used antibodies against the following proteins: EGFP (1:200; Molecular Probes), NKCC2 (1:2000; Shimadzu), AQP2 (1:200; Abcam, Cambridge, Science Park, UK), and sodium-hydrogen exchanger 3 (NHE3; SLC9A3P2) (1:300; ACR). The antibody against NHE3 was used in lieu of the antibody against NPT2a, due to a lack of the latter's availability. We immunostained the deparaffinized sections using the avidin-biotin complex immunoperoxidase method.

For transmission confocal microscopy (LSM510, Carl Zeiss, Jena, Germany), frontally sectioned kidney slices were frozen in liquid N_2_, embedded in Optimal Cutting Temperature compound (Sakura Finetek, Tokyo, Japan), and stored -80°C until use. The same antibodies as described above were used, and nuclear staining was done with Hoechst 33342 (Cat# H3570, Thermo Fisher, MA, USA).

## Results

### Design of Nkcc2 promoters

**Fig 1:**
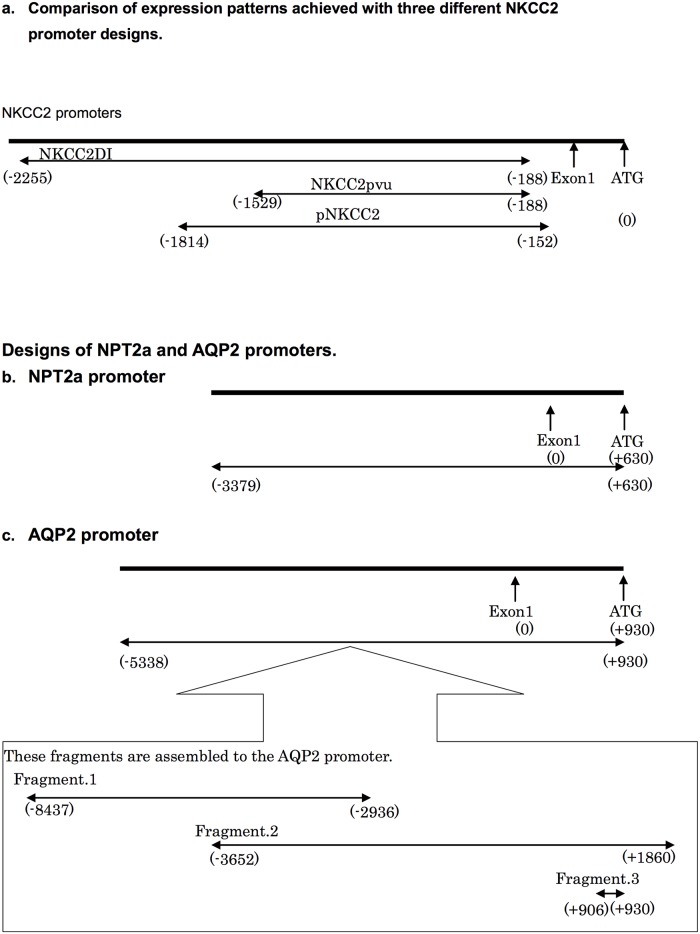
Designs of cloned pNKCC2, NPT2a, and AQP2 promoters. a. Three different NKCC2 promoters used in this study. Transgene expression patterns were compared among the constructs, using these promoters. b. NPT2a promoter. c. AQP2 promoter.

To optimize the efficiency of the promoter constructs, we first compared three different promoters of *Nkcc2*. These gene constructs for the 5′ upstream region of the *Rattus Nkcc2* (*Slc12a1)* genes were prepared according to the data on construct-expression relationships previously published [18]. The three promoters, named pNKCC2DI, pNKCC2pvu, and pNKCC2, are positioned as shown in Fig 1a. Using primary cultured tubular cells, Igarashi *et al*. found that pNKCC2pvu exhibited the highest level of promoter activity, while pNKCC2DI exhibited the lowest level of activity, among the seven promoters examined [18]. However, the sequence of area A only harbored by pNKCC2DI (Fig 1a) was found to have conserved sequences across various mammalian species, and was also found to have many transcription binding sites, such as c-Rel, CREB, and Evi-1. For this reason, we expected to see better gene expression using pNKCC2DI than the other constructs. We then linked the respective promoters to the EGFP gene to evaluate promoter-EGFP expression patterns.

### Comparison of administration routes

Four different approaches of adenoviral infection were compared by observing adenovirus-derived EGFP expression in the kidneys. Because approaches (b), (c), and (d) in S1 Fig did not show satisfactory expression patterns of NKCC2 in the rat kidney, we selected approach (a) for further experiments. The (a) procedure was, the renal artery flushed out with 1ml of natural saline, the adenovirus vectors of 10^8^U/ml, 1ml are injected into the renal artery, the renal artery was clamped for 10 min to enhance infection efficiency, and the last, the clamps were removed from the vessels to restore blood flow in rats.

The explanations for this decision are the following. Approach (b) was continuous slow drip for 16 h into the left renal artery: But there was no promoter-driven EGFP detected; we speculate the infection rate of adenovirus in this case was very low, due to the presence of the blood. Approach (c) was to inject into the urinary tract to flow upstream: The promoter-driven EGFP was stained very clearly; however, the microvilli of the proximal tubule vanished; we suspect this was due to the excessive intra-tubular pressure by retrograde flow. This method not only injured the kidney structure, but also introduced a risk of urinary tract infection. Approach (d) was direct injections to the renal parenchyma from all directions: The EGFP expression area was restricted to be solely within the injection sites, and the structure of renal parenchyma was damaged by repeated injections.

**Fig 2:**
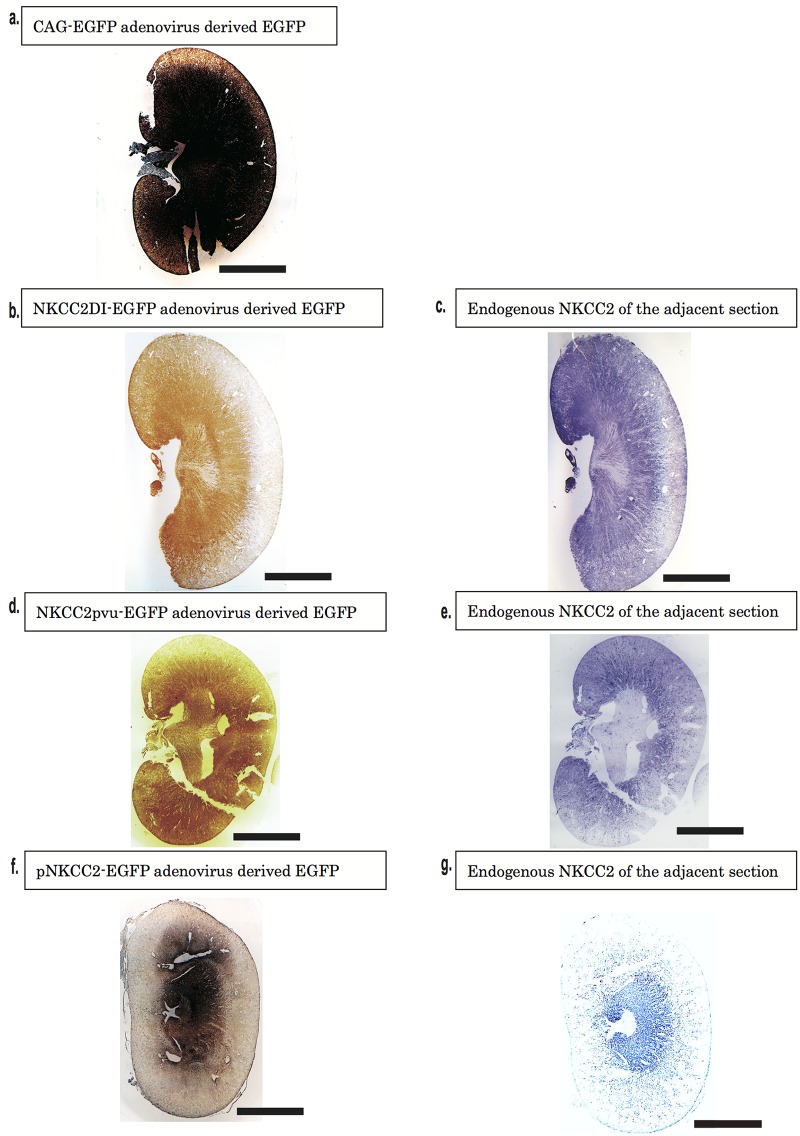
Immunostaining of the kidneys from adenovirus administered rats. a. EGFP staining of the kidneys in rats received CAG-EGFP adenovirus (brown). b and c. EGFP staining of the kidney in rats received NKCC2DI-EGFP (b, brown). The adjacent section of the kidney was stained with endogenous NKCC2 (c, blue). d and e. EGFP staining of the kidney in rats received NKCC2 pvu-EGPF adenovirus (d, brown). The adjacent section was stained with NKCC2 (e, blue). f and g. EGFP stating of the kidney in rats received pNKCC2-EGPF adenovirus (f, brown). The adjacent section was stained with NKCC2 (g, blue). Scale bar: 1 cm.

Using intrarenal volus injections of adenovirus (a), we tested for differences in NKCC2 expression. Non-specific expression of CAG-EGFP was evident throughout the kidney tissues, suggesting the uniform infection of adenovirus and the distinct expression of the gene construct, but without site specificity (Fig 2a).

We next compared the differences in expression patterns of the three promoters of *Nkcc2*, using the endogenous expression of NKCC2 as a reference. Among the three promoters, the expression patterns of NKCC2DI-derived EGFP (Fig 2b) most closely matched the staining patterns of the endogenous NKCC2 protein (Fig 2c). By contrast, NKCC2pvu showed broader distribution (Fig 2d) than endogenous NKCC2 (Fig 2e), also, pNKCC2-derived EGPF showed broader distribution than endogenous NKCC2 (Fig 2g).

**Fig 3:**
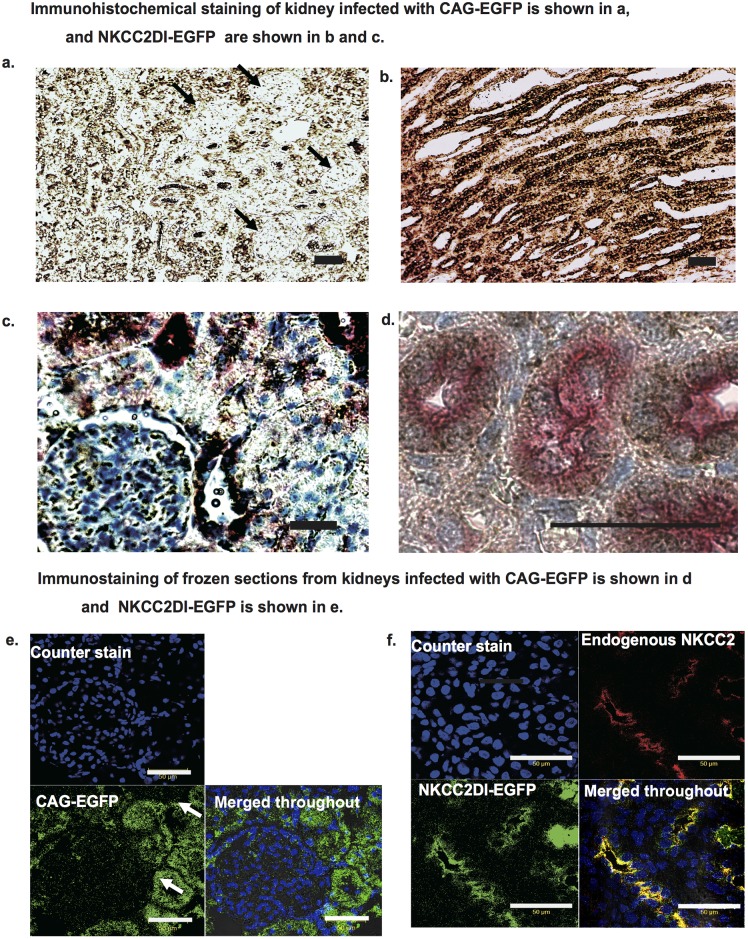
Immunohistochemistry of the kidneys. a. Paraffin-embedded sections of the kidneys infected with CAG-EGFP adenovirus. The arrow indicates glomerulus. Adenovirus-derived EGFP was stained in brown. b and c. Paraffin embedded sections of the kidney infected with NKCC2DI-EGFP adenovirus. Adenovirus-derived EGFP is shown in brown and endogenous NKCC2 is shown in pink. e and f. Immunostaining of frozen kidney sections infected with CAG-EGFP is shown in e and NKCC2DI-EGFP is shown in f. e.Green indicates CAG promoter derived EGRP and f. Green indicates NKCC2 promoter-derived EGFP. f. Red indicates endogenous NKCC2. e and f. Blue indicates nucleus.

At higher magnification, CAG-EGFP was expressed throughout the kidney tissue, except for the glomeruli (Fig 3a and 3b), where it was not expressed. Similar to observations made at lower magnification, staining of pNKCC2DI-EGFP and endogenous NKCC2 localized to the same cells. Double staining was peformed to verify the precise pattern of protein expression. The expression of pNKCC2DI-EGFP (brown color) was localized to the TALH cells, and endogenous NKCC2 protein (pink color) was localized to the same TALH cells (Fig 3c and 3d). To further verify these specific expression patterns, we performed confocal microscopy and confirmed that pNKCC2DI-EGFP and endogenousNKCC2 were expressed in the TALH cells (Fig 3f). By contrast, CAG-EGFP stained in the cytoplasmic areas of all types of kidney tubules non-specifically (Fig 3e). Conversely, we did not detect EGFP expression in the contralateral kidney, liver, spleen, ileum, or lung via any of the four injection routes used.

Regarding adverse effects or toxicity induced by intra-renal arterial injections of adenovirus, apparent damage was not observed in the renal tissue structure by microscopic examination, and there was no change in renal and liver function by laboratory serum and urine data.

### Npt2a promoter for targeting proximal tubules

Next, the 5′ upstream region of the *Rattus Npt2a (Slc34A)* gene, encoding the NPT2a promoter, was constructed. We designed our promoters based on the premise that an optimal promoter would begin at the -1 codon from the ATG start codon of the 5′-flanking region, and that the longer the promoter was, the better the expression would be (Fig 1b and 1c).

**Fig 4:**
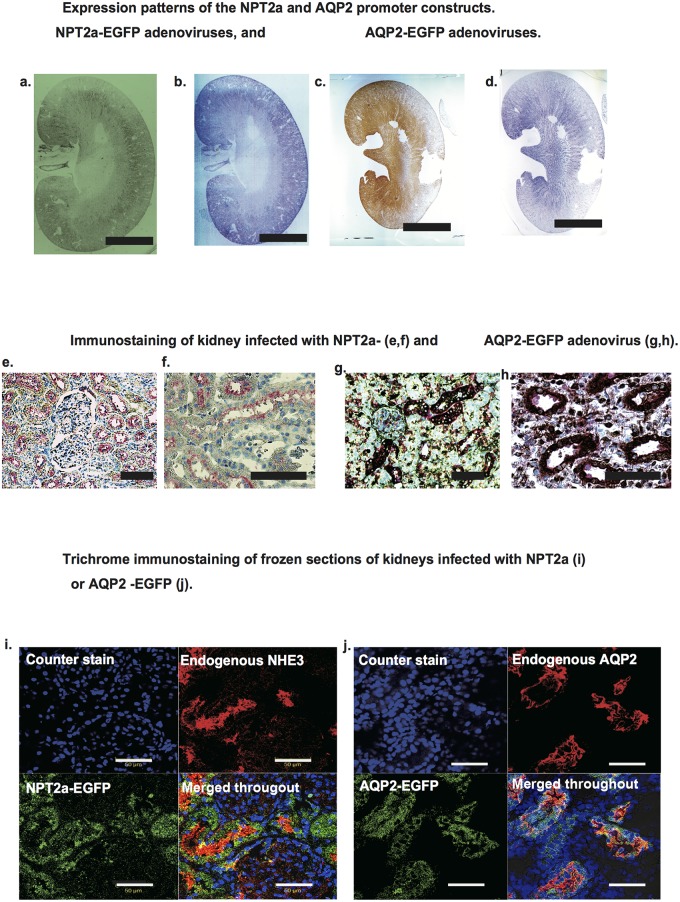
Expression patterns of the NPT2a and AQP2 promoter constructs. a and b. Paraffin embedded sections. a. NPT2a adenoviruses derived EGFP was shown in brown (a). Endogenous NPT2 was stained in the adjacent section (b, shown in blue). c and d. AQP2-adenovirus derived EGFP was shown in brown (c), and endogenous AQP2 was shown in blue in the adjacent section (d). e and f. Immunostaining of kidney infected with NPT2a-EGFP. Brown indicates adenovirus-derived EGFP, and pink indicates endogenous NHE3. Nucleus is stained in blue. g and h. Immunostaining of kidney infected with AQP2-EGFP. Brown indicates adenovirus-derived EGFP, and pink indicates AQP2. Nucleus is stained in blue. i and j. Immunostaining of frozen sections of kidneys infected with NPT2a (i) or AQP2-EGFP (j). Green indicates adenovirus-derived EGFP and blue indicates nucleus. Red indicates endogenous NHE3 (i) or AQP2 (j).

Since an effective and specific antibody against NPT2a was not available, an antibody against NHE3 was used to identify proximal tubule cells. Under low magnification, the expression patterns of NPT2a-EGFP and endogenous NHE3 looked similar (Fig 4a and 4b). Moreover, double staining was peformed with NPT2a-EGFP (brown color) and NHE3 protein (pink color) to determine the precise expression in the same cells. However, NPT2a-EGFP (brown color) localized to the cytoplasm of the proximal tubule cells, whereas NHE3 protein (pink color) localized to the apical membrane of the proximal tubule cells (Fig 4e and 4f). Laser confocal microscopy further confirmed that NPT2a-EGFP was localized to the cytoplasm, while endogenous NHE3 was localized to the apical membrane of the identical cells (Fig 4i). To explain this in more detail, both NPT2a protein and NHE3 protein were shown in the cells of the proximal tubule, but their localization patterns within those same cells are different. It is probably because NPT2a promoter driven EGFP and NHE3 promoter driven NHE3 (endogenous NHE3) are different protein and different distribution pattern in the same cells because of different posttranslational modifications. Our aim here is to demonstrate that while NPT2a-derived EGFP and endogenous NHE3 appear in the same cells, but the same expression pattern in the cells. Of note is that the expression site of targeted transgene is the present study was clearly restricted to the authentic tubule segment without aberrant expression in other segments.

### Aqp2 promoter for targeting collecting ducts

We constructed the 5′ upstream region of the *Rattus Aqp2* gene, encoding an AQP2 water channel promoter. At low magnification, AQP2-EGFP and endogenous AQP2 were detected in the same areas (Fig 4c and 4d). Under high magnification, AQP2-EGFP (brown) staining was localized to the cytoplasm of collecting duct cells, whereas endogenous AQP2 (purple) staining was limited to the apical membrane of the collecting duct cells (Fig 4g and 4h). These findings were verified by laser confocal microscopy (Fig 4j). Again, the expression site of targeted transgene in the present study was restricted to the authentic tubule segment without aberrant expression in other segments.

## Discussion

This study clearly demonstrated that site-specific expression of an exogenous protein is possible by adenovirus-mediated transgene methodologies. We chose an adenovirus vectors as the gene carrier because adenoviral vectors exhibit the highest levels of transgene expression among all currently available vectors [9, 11–14, 19]. Moreover, adenoviral vectors have an important advantage in that they are able to deliver large-sized genes (up to 8 kbp) compared to other viral vectors [8]. In addition, the adenoviral vector infects quiescent or terminally differentiated cells, i.e., both dividing and non-dividing cells, and therefore has considerable versatility for gene induction within contexts such as airway epithelial cells [20]. To develop targeted gene therapeutics, and to yield a clear phenotype, sufficient amounts of expressible transgenes are necessary. We attempted to develop specific promoters that harbor the -1 position of the translation initiation codon, and with as lengthy a promoter as possible. In this study, we succeeded in making constructs as long as 4–8 kbp, leading a successful expression of the transgenes. Several routes of administering the adenovirus vector were compared, ranging from direct infusion into the renal artery to retrograde infusion through bladder; only the method of direct infusion into the renal artery demonstrated successful adenoviral infection in kidney tissues. The other approaches did not work well. In previous reports, retrograde ureter infusion was shown to be amenable to transgene methodologies [20, 21]. However, in our hands, retrograde ureter infusion did enable pNKCC2DI-EGFP to be expressed in TALH cells, but tubule structures were badly damaged, probably as a result of the increase in intra-tubular pressure. The method of infusing adenovirus into the renal artery for 16 h did not work well; we speculate it is because the blood plasma components have immune-mediated mechanism, may inhibit the virus from infecting the cells. Thus, we conclude that washing out the blood from the vessels for a while and intra-renal artery injection of high concentration of adenoviruses may be indispensable for successful infection of the renal cells with adenoviruses. Then, no detectable damages were found in the renal tissue. Considering of the mean width of the slit diaphragm of the glomerulus is less than the diameter of the adenovirus, the adenovirus might reach tubules through the vasculature but glomerulus.

There was slight difference in expression patterns between promoter-driven EGFP and endogenous protein. The reason was estimated because of the difference of the expressing protein. NPT2a, NPCC2 and AQP2 proteins have their own posttranslational modification systems and distribution pattern in the cells, these patterns are different from those of EGFP. To be more specific, it can be explained by the difference of posttranslational modifications, i.e. trafficking signal peptides [23] and/or a shortage of posttranslationally modifiable sites, such as those that could undergo phosphorylation upon activation [24]. Thus, the merged picture of this protein's expression patterns showed different localization to both the membrane and cytoplasm. Anyway, the EGFP expression site in the present study was restricted to the authentic tubule segment without aberrant expression in other segments. These results indicate that promoter-directed gene expression were successfully accomplished

This study indicates the future potential for the clinical application of gene therapy for certain kidney diseases, including tubular disease. Inherited and acquired tubular diseases encompass a variety of kidney conditions such as Bartter syndrome, Gitelman syndrome, Fanconi syndrome, Liddle syndrome, nephrogenic diabetes mellitusand diabetes insipidus. Of special note is that these tubular diseases all involve transporter dysfunction in specific renal tubules. Thus, the final goal of successful site-directed treatment requires site-specific expression to the target cells. Therefore, further studies of the translation of coding regions should follow in the near future. The targeting of specific cells, as demonstrated in this study, may open new horizons for site-directed gene therapy—even in the complex tissues of the kidney. Adenovirus infusions to the target tubular cells would be accomplished with percutaneous catheter manipulation in human.

We must mention several limitations to the present study. Firstly, the translation of the target protein has not achieved in this study, and this step is fundamental to making gene therapy amenable to clinical use. Secondly, the adenovirus gene expression is not permanent because adenovirus is not integrated.

Despite these limitations, this is the first study to report successful targeting of gene expression to specific sites of the kidney tubules. Future work is necessary to advance site-specific gene therapy, and the present study may serve as the first step.

## Conclusions

Transducing genes into the kidneys, and in a cell-selective manner, have been accomplished by the combination of two strategies. The first was the cloning of appropriate, site-specific promoters that have sufficient specificity and expression efficiency. The second was adenovirus infection through direct injection into the renal artery after washout the blood in the kidney. Although the translation of target proteins in the specific site remains the next required step, the methods described in the present study probably make site-directed gene therapy much easier than was ever thought possible.

## Supporting information

S1 Fig Gene-targeting strategy to the specific cells in the kidney. Four routes of adenovirus infection were compared: 1) bolus injection for 2 min into the left renal artery, 2) continuous slow drip for 16 h into the left renal artery, 3) injection into the urinary tract to flow upstream, and 4) direct injections to the renal parenchyma from all directions.
(TIFF)
