# Supplementary figures and images for: Targeting gene expression to specific cells of kidney tubules in vivo, using adenoviral promoter fragments
Source: PLoS One. 2017 Mar 2;12(3):e0168638. doi: 10.1371/journal.pone.0168638 (PMC5333796; doi:10.1371/journal.pone.0168638)

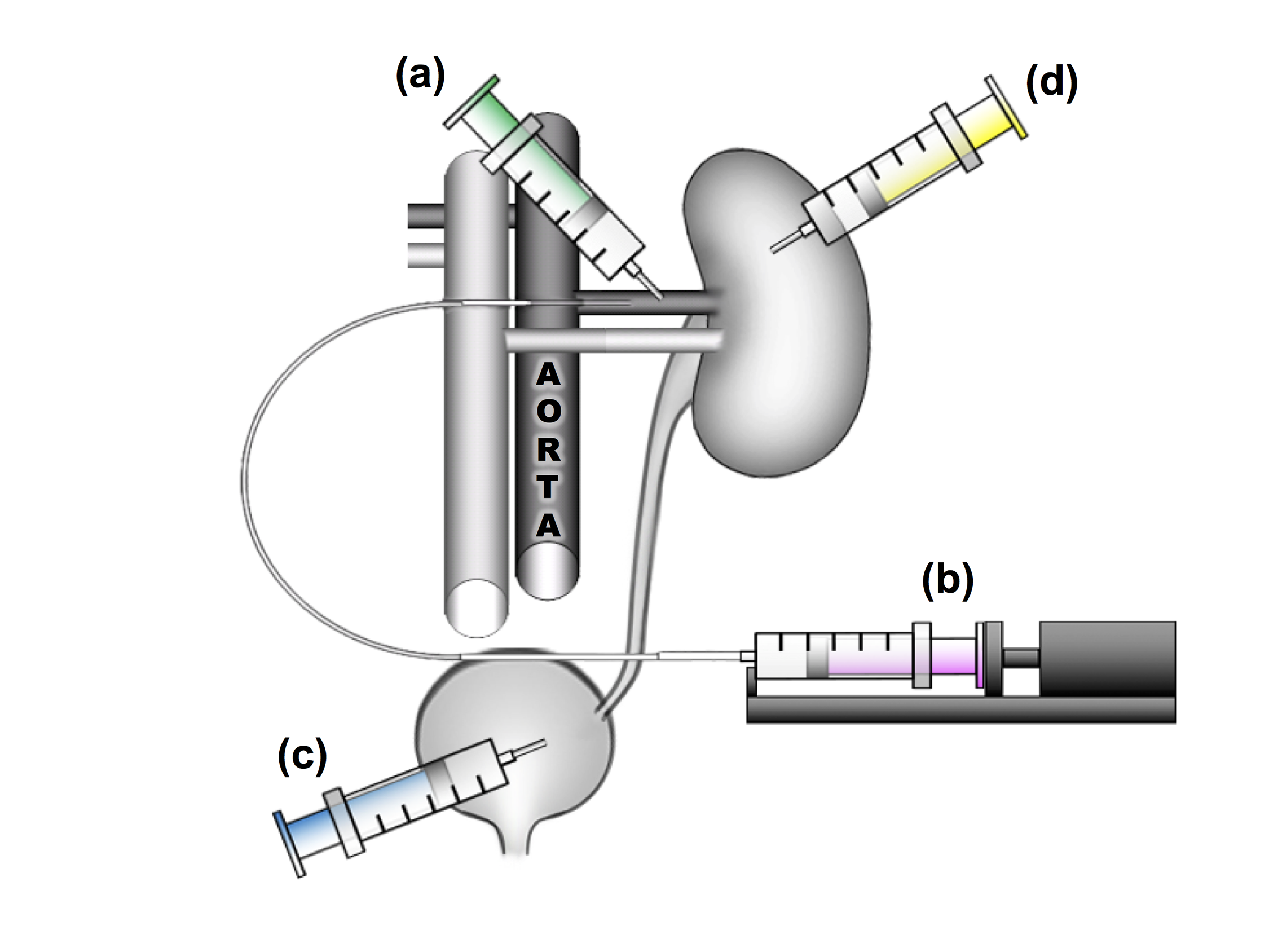

Supplement: S1 Fig — Four routes of adenovirus infection were compared: 1) bolus injection for 2 min into the left renal artery, 2) continuous slow drip for 16 h into the left renal artery, 3) injection into the urinary tract to flow upstream, and 4) direct injections to the renal parenchyma from all directions. (TIFF) [file pone.0168638.s001.tiff]
